# Biophysical Aspects of T Lymphocyte Activation at the Immune Synapse

**DOI:** 10.3389/fimmu.2016.00046

**Published:** 2016-02-15

**Authors:** Claire Hivroz, Michael Saitakis

**Affiliations:** ^1^Institut Curie Section Recherche, Paris, France; ^2^INSERM U932, Paris, France; ^3^PSL Research University, Paris, France

**Keywords:** T lymphocytes, immune synapse, force control, TCR, LFA-1, biomechanics, stiffness

## Abstract

T lymphocyte activation is a pivotal step of the adaptive immune response. It requires the recognition by T-cell receptors (TCR) of peptides presented in the context of major histocompatibility complex molecules (pMHC) present at the surface of antigen-presenting cells (APCs). T lymphocyte activation also involves engagement of costimulatory receptors and adhesion molecules recognizing ligands on the APC. Integration of these different signals requires the formation of a specialized dynamic structure: the immune synapse. While the biochemical and molecular aspects of this cell–cell communication have been extensively studied, its mechanical features have only recently been addressed. Yet, the immune synapse is also the place of exchange of mechanical signals. Receptors engaged on the T lymphocyte surface are submitted to many tensile and traction forces. These forces are generated by various phenomena: membrane undulation/protrusion/retraction, cell mobility or spreading, and dynamic remodeling of the actomyosin cytoskeleton inside the T lymphocyte. Moreover, the TCR can both induce force development, following triggering, and sense and convert forces into biochemical signals, as a *bona fide* mechanotransducer. Other costimulatory molecules, such as LFA-1, engaged during immune synapse formation, also display these features. Moreover, T lymphocytes themselves are mechanosensitive, since substrate stiffness can modulate their response. In this review, we will summarize recent studies from a biophysical perspective to explain how mechanical cues can affect T lymphocyte activation. We will particularly discuss how forces are generated during immune synapse formation; how these forces affect various aspects of T lymphocyte biology; and what are the key features of T lymphocyte response to stiffness.

## Introduction

T lymphocytes are motile small cells, which play a key role in adaptive immune responses against pathogens and tumor cells. T lymphocyte activation is triggered by the recognition *via* the T-cell receptor (TCR) expressed at the surface of T lymphocytes, of antigenic peptides, derived from pathogens or tumors and associated with major histocompatibility complex (MHC) molecules exposed at the surface of antigen-presenting cells (APCs). Numerous costimulatory or co-inhibitory receptor/ligand pairs present at the plasma membrane of both cells can also modulate T lymphocyte activation (1). Thus, T lymphocyte activation is crucially dependent on the close interaction between both plasma membranes. This interaction is organized in time and space by the formation of structures, termed immune synapses, in which molecules are unevenly distributed and segregated while remaining mobile (2–4).

Thanks to increasingly sophisticated visualization techniques, more and more information is accumulated on the organization of both plasma membrane receptors and signaling molecules at the immune synapses. Visualization of T lymphocyte interactions with APCs showed that these cellular partners were submitted to pulling, pushing, and shearing forces due to cell motility relative to each other (5); continuous spontaneous motion of plasma membrane (6); and cytoskeletal remodeling (7–9). A specific function of mechanical forces in T lymphocyte activation was even proposed in the first study showing the dynamic formation of immune synapse (10). Forces exerted by T lymphocytes during these contacts have only been quantified recently (11–13). The TCR itself was shown to be a mechanosensor, i.e., able to convert the mechanical forces exerted during TCR binding to peptide–MHC complexes into a biochemical signal (14–16). Finally, at the resting T lymphocyte membrane, there are organized complexes of receptors and signaling molecules, maintained in a state of basal activity where the membrane receptors are readily available to interact with their ligands on an APC surface and to induce a signaling cascade. This dynamic organization resembles a buffer condition that is able to respond to a minute amount of agonist pMHC in a sea of endogenous pMHCs and is optimized not only for the identification of antigen but also for the initiation and amplification of signals following successful antigen recognition (17–19).

Although formation of the immune synapse has been extensively studied, information on the mechanical properties of the microenvironment and on how these properties affect T lymphocyte functions has only recently become available. We will thus review herein recent advances on the knowledge of how T lymphocytes generate or respond to forces during antigen recognition and immune synapse formation.

## Forces in T Cells

When interacting with an APC, T lymphocyte morphology changes drastically: the cell moves on the APC surface, develops invadosome-like structures which push into the cortex of the APC (20–22), spreads on the APC, and eventually stops. During each of these steps, T lymphocytes exert and/or are submitted to forces, which can affect receptor/ligand bonds. We will discuss the pathways involved in the generation of these forces.

### Spontaneous Membrane Oscillations and Formation of Protrusions

**Figure 1:**
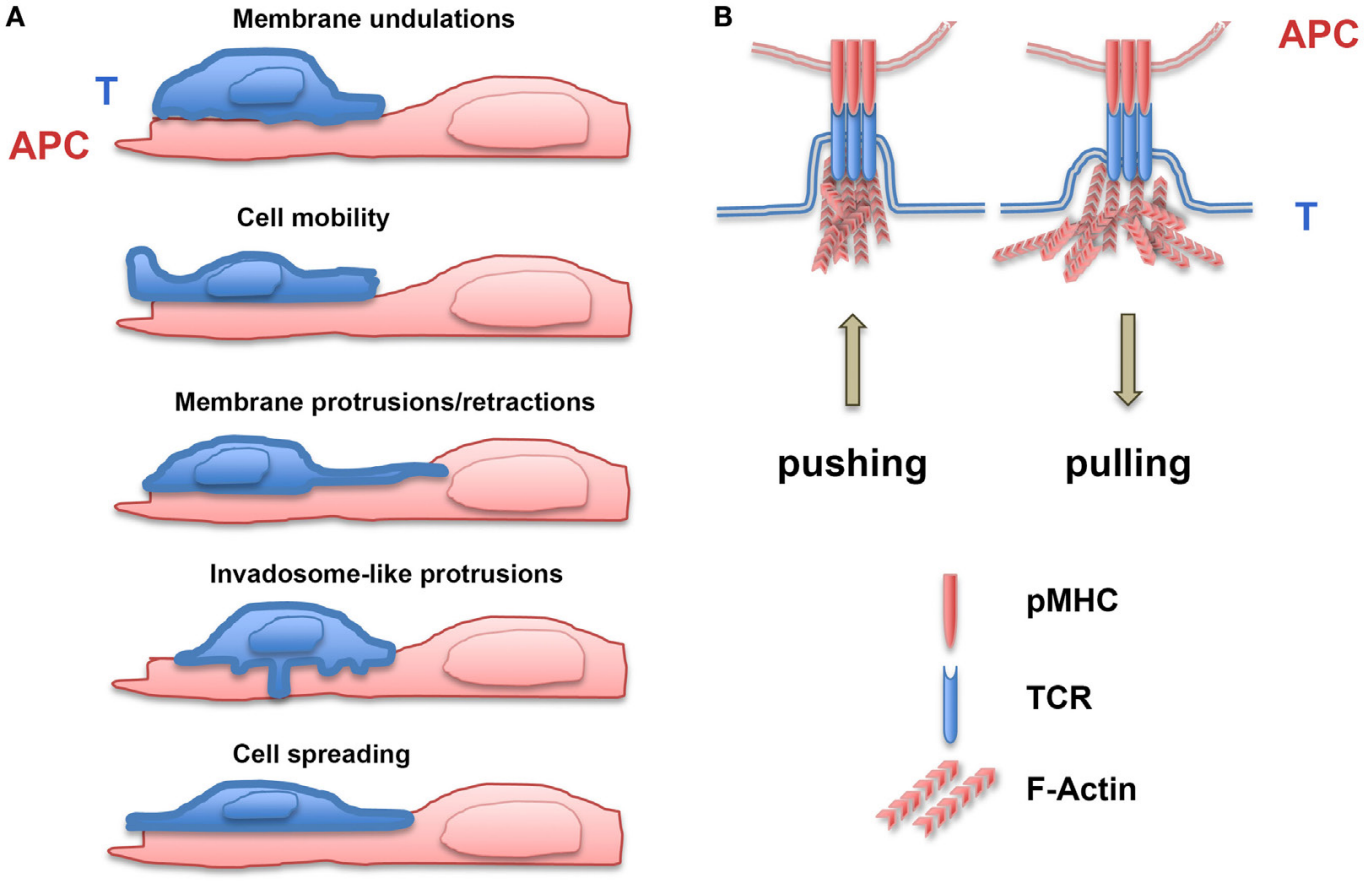
**Generation of forces during T lymphocyte/APC contacts**. **(A)** Forces are exerted on receptor/ligand bonds by membrane T lymphocyte undulations, cell mobility, membrane protrusions/retractions, invadosome-like protrusions, and cell spreading on antigen-presenting cells (APC). **(B)** Upon TCR triggering, T lymphocytes develop pushing and pulling forces on TCR/pMHC bonds, which depend on polymerization of F-actin.

Lymphocytes, such as other cell types, display membrane undulations with amplitude of several tens of nanometer and frequency ranging between 0.2 and 30 Hz (23). Moreover, when interacting with a surface, T lymphocytes rapidly develop protrusions and retractions that are organized in lateral waves along the cell membrane (24). Filopodia or microvilli are protrusive structures with a length between 0.1 and several micrometers that display receptors at their tips and present cycles of protrusions/retractions, which allow them to sense both the mechanical and biochemical environments (25). In order to grow, filopodia have to develop protrusion forces against the membrane that are mainly produced by actin polymerization at the filopodial tip (26–28). Filopodial diameter is in the same range as the diameter of membrane tubes, the generation of which requires forces ranging from 5 to 30 pN (29). Filopodia not only exert protrusive/pushing forces but also retracting/pulling ones, which have been measured using traction force microscopy, i.e., by recording the local deformation of a soft substrate of known stiffness in which fluorescent beads are embedded (29, 30). In neuron cells, the pulling forces developed by filopodia have been shown to be in the order of 1 nN (31). During their migration on endothelial cells, T cells can form F-actin-based protrusions, termed invadosome-like protrusions (ILPs) (32). These structures are small (diameter of ~0.2 μm) and transient (half-life of ~20 s) and physically push against the endothelial cell surface (20, 33). It has been postulated that ILPs can sense the stiffness of endothelial cells by “tiptoing” their surface (32, 34, 35). More recently, Yang et al. (36) described the forces developed by chemotactic T lymphocytes. A laser trap was used to position two beads, one as source of chemokine gradient and the other to measure the forces exerted by the migrating T lymphocytes (in their case, the Jurkat leukemic T cell line). The protrusion forces measured at the leading edge of Jurkat cells migrating in a gradient of SDF-1 were as high as 1000 pN and increased in parallel to the chemoattractant gradient. Moreover, the forces required to stop the migrating cells ranged from 100 to 300 pN. Finally, tensile forces may also be present at the membrane when short molecule bridges at the T lymphocyte/APC interface (i.e., TCR/pMHC or CD2/CD58 pairs) exclude other larger molecules (LFA-1/ICAM-1 pairs or CD45) (37). These results (see Figure 1 for summary) demonstrate that spontaneous undulations of the T lymphocyte membrane and formation/retraction of filopodia and other cellular protrusions can generate forces that facilitate probing of the biomechanical microenvironment (38). Meanwhile, receptor/ligand bonds are submitted to a wide range of forces during cell migration. This will result in the modulation of signaling cascades induced by mechanosensitive molecules.

### TCR Engagement Induces Force Generation

Despite the increasing knowledge of signaling pathways engaged after recognition of pMHC by the TCR, the triggering mechanism of the signaling cascade still remains controversial (39). Several mechanisms have been proposed, which involve aggregation, conformational changes, and segregation (40). This matter has been reviewed extensively and will not be addressed further. Yet, studies aiming to investigate if and how receptor engagement generates forces that might then be converted in biochemical signal are sparse.

In one study, Hosseini et al. used atomic force microscopy (AFM) to measure the adhesion forces between a T cell hybridoma and a B cell line used as APC (41). Results showed that in the presence of antigen, adhesion forces built up with time of conjugate formation, starting from 1 to 2 nN at the beginning of the interaction to 14 nN after 30 min. The adhesion forces were mainly due to lymphocyte function-associated antigen-1 (LFA-1)-mediated adhesion, since the integrin inhibitor BIRT377 almost completely abolished forces in the conjugates (41). Similar experiments were performed on conjugates formed between mouse primary T lymphocytes expressing the OT1 transgenic TCR and a mouse dendritic cell line presenting OVA peptides of different affinities (42). In this experimental model, adhesion forces between cellular partners were smaller (up to 1.5 nN) and correlated to the ability of the different peptides to activate the T lymphocytes; better agonist peptides induced stronger adhesion forces (42).

Even though the above studies provided values for interaction forces between T lymphocytes and APCs, they could neither address the question of the relative contribution of different molecules to the forces measured nor the question of the contribution of each cell partner in force generation. Therefore, we adapted the biomembrane force probe (BFP) technique, which was developed to probe molecular adhesion (43), in order to assay the generation of forces by T lymphocytes. The BFP consisted of a red blood cell (RBC), which was on one side coupled to a bead coated with antibodies and held on the other side by a pipette. A human primary CD4^+^ T lymphocyte held by a second pipette was brought into contact with the BFP. Activation of the T lymphocyte was monitored by imaging increases in the intracellular Ca^2+^ concentration, [Ca^2+^]i, and forces exerted by the T lymphocyte on the BFP were measured on time lapse stacks of images by determining the elongation of the RBC with respect to the position of the fixed micropipette (11). When the bead was coated with anti-CD3 antibodies, three consecutive phases were observed following T lymphocyte contact with the BFP: a latency phase, which lasted less than a minute during which no force and no [Ca^2+^]i increase were observed; a pushing phase consisting of the growth of a directional cell protrusion characterized by an initial axial compression of the RBC and a peak in [Ca^2+^]i increase; and, in most cases, a pulling phase characterized by protrusion/retraction and generation of pulling forces, as witnessed by the elongation of the BFP. The initial forces exerted by T lymphocytes on the RBC were around 25 pN for a probe stiffness of 50 pN/μm. Measurement of elongations showed that CD3 engagement on T lymphocytes triggered a constant pulling loading rate of ~2 pN/s. These characteristic three phases were not observed when the bead was coated with anti-MHC-I antibodies, showing that the mere binding of the bead to the T lymphocyte membrane is not sufficient to induce forces (11). Engagement of LFA-1 together with CD3 modified the forces exerted compared to CD3 alone: when the bead coupled to the BFP was coated with an anti-CD18 antibody (specific against the β_2_ chain of LFA-1), a clear decrease in growth velocity and protrusion length during the pushing phase was observed. Moreover, the pulling phase started earlier and the protrusion morphology was changed from a “tube-like” to a “cup-like” structure resembling the phagocytic cup. Engagement of LFA-1 alone on resting primary T lymphocytes did not generate any pushing phase. It also generated 100-fold lower pulling loading rates (0.2 pN/s for a probe stiffness of 50 pN/μm) than the pulling loading rates induced by CD3 engagement alone (25 pN/s for the same probe stiffness). Absence of force generation in response to just LFA-1 triggering can be attributed to the fact that T lymphocytes were not pretreated for inside-out signaling induction (i.e., pretreatment with chemokines, anti-CD3, or phorbol-ester). Thus, integrins can generate traction forces and modify the forces induced upon CD3/TCR triggering. Indeed, force measurements performed on human neutrophils submitted to chemotactic gradients on hydrogel substrates revealed that neutrophils also generated traction forces, which were dependent on β_2_ integrin engagement and signaling (44). This was not specific to LFA-1 engagement since binding of α_5_β_1_ integrins to fibronectin and activation of these integrins by addition of Mn^2+^ were also been shown to induce traction forces (45).

Two more studies confirmed that T lymphocytes generate significant forces upon CD3 engagement. In the first one, Bashour et al. used elastomer pillar arrays of known spring constant coated with activating antibodies (12). In this experimental setting, each pillar tip deflection caused by cell attachment and spreading is monitored using live cell videomicroscopy (46, 47). Human primary CD4^+^ T lymphocytes were put on micropillars coated with anti-CD3 antibodies and several phases were observed (12). In the first phase, cell spreading generated only minor forces. After this phase, cells ceased to spread and started to exert significant traction forces, which were essentially centripetal and exerted mostly at the cell periphery. The forces generated per pillar were around 50 pN. In the same study, forces exerted by mouse primary CD4^+^ T lymphocytes on the same pillars were fourfold higher (200 pN/pillar). No forces were measured when pillars were coated with an antibody against the costimulatory molecule CD28 alone. However, the dual presence of anti-CD3 and anti-CD28 antibodies resulted in doubling the traction forces exerted by T lymphocytes on the micropillars. This was observed when the anti-CD28 Ab was present on the pillar together with anti-CD3 or when added in solution (12). These results suggest that the traction forces induced by CD28 engagement are not directly generated through the CD28 receptor. They are rather due to signaling-dependent amplification of the forces triggered by TCR engagement.

In another study, Hui et al. used traction force microscopy to measure the forces exerted by Jurkat cells during TCR activation (13). Jurkat cells were put on polyacrylamide gels coated with anti-CD3 antibodies and embedded with fluorescent beads at the top surface. The traction forces exerted by the cells were measured by tracking fluorescent bead displacement. In the presence of anti-CD3 and for a substrate stiffness rigidity of 1–2 kPa, traction forces were in the order of 2 nN, whereas forces exerted on substrates coated with a non-activating antibody were below 1 nN (13).

From the above results, it is evident that TCR–CD3 engagement can generate forces in T lymphocytes. These forces can be modified by the engagement of costimulators, such as LFA-1 and CD28. We will now discuss the potential outcomes of forces on T cell activation.

## Effect of Forces on T Cell Activation

We have seen in the previous paragraphs that membrane undulations, protrusions and retractions, cell migration, and TCR triggering can generate forces that can be exerted on receptor/ligand bonds. In the next section, we will discuss the effect of these forces on specific receptor/ligand pairs at the T lymphocyte/APC interface, i.e., TCR/pMHC and LFA-1/intercellular adhesion molecule-1 (ICAM-1), and on overall T lymphocyte activation.

### Forces Exerted on TCR/pMHC Bonds

T lymphocytes typically recognize peptides of 8–11 amino acids presented by MHC molecules. The TCR can “sense” a single amino acid substitution and translate it in a different functional response. Moreover, T lymphocytes can precisely discriminate a small number (2–10) of pMHC complexes for which they are specific within a sea of self or foreign peptide-MHC molecules (17–19). How this exquisite specificity and sensitivity is achieved is still a matter of investigation. Forces exerted on the TCR–pMHC bonds may have a key role in these processes. Indeed, it has been shown that the TCR functions as a mechanosensor, i.e., it can convert mechanical cues into biochemical signals (16). The first direct evidence was obtained by E. Reinherz’s group, who used optically trapped beads coated with non-activating anti-CD3ϵ antibodies or pMHC to apply forces on the TCR and monitored T lymphocyte activation by measuring [Ca^2+^]i increase (14). They showed that T cells were triggered mechanically, since application of a tangential force (50 pN) to the coated bead induced calcium signaling. Force application on beads coated with pMHC complexes that did not bind TCR had no effect on calcium flux. In another study, Li et al. used a fibroblast cell line expressing a single chain Fv anti-CD3ϵ antibody elongated by a tether (15). Binding to CD3 did not induce calcium signaling unless a mild perpendicular shear stress or a normal pulling force on the T lymphocyte bound to the surrogate APC was applied (15). By contrast, pulling forces applied on CD28 or CD62L did not increase intracellular calcium levels. These studies demonstrated that the TCR could transform a mechanical signal (force) into a biochemical one ([Ca^2+^]i increase). Yet, several questions still remain unresolved and particularly whether forces applied on TCR/pMHC bonds can affect T cell antigen recognition and discrimination. Work from the group of C. Zhu elegantly demonstrated that mechanical forces applied using a BFP on TCR/pMHC-I (48) and TCR/pMHC-II (49, 50) affected dissociation kinetics in a peptide-specific way. Forces applied to the bonds prolonged the lifetimes of single TCR–pMHC bonds for agonists (catch bonds) but shortened those for antagonists (slip bonds). When forces of 10 pN were applied by BFP on OT1 TCR/pMHC bonds, the ratio of OT1 TCR–pMHC bond lifetimes for the agonist peptide versus a weaker altered peptide grew 57-fold compared to when no force was applied (49, 50), demonstrating that forces can increase the power of antigen discrimination. The functional outcome of different peptides recognized by the same TCR was also shown to be coupled with the cumulative lifetime of the TCR–pMHC bonds (49).

A TCR deformation model where mechanical stress could induce conformational changes that would unmask sites of phosphorylation and allow TCR signaling was also proposed (51). Application of forces on the TCR would expose the immunoreceptor tyrosine-based activation motifs present in the CD3ϵ and ζ chains, otherwise buried into the hydrophobic core of the membrane lipid bilayer (52, 53). More recently, it was proposed that the structural features of TCR–CD3 complexes are adapted to permit sensing and discrimination of the forces to which TCR/pMHC bonds are submitted (54). Das et al. used optical tweezers and DNA tether spacer technology, which allow for application of forces in the order of piconewton with a spatial precision of nanometer, in order to address the mechanisms involved in the control of strength and lifetime of the TCR–pMHC-I bonds (55). They confirmed that forces applied on TCR–pMHC-I bonds increased the lifetime of the bond and showed that the state of the CβFG loop region, a 12-amino acid peptide present in the constant region of the β chain of the TCR of all mammalian αβTCRs (56), is involved in the increased lifetime of TCR–pMHC-I bonds submitted to tensile forces (55). This study suggests that forces physically modify the αβTCR by switching it from an “extended form” that binds weakly to a “compact form” that binds more robustly. The conformational changes of the TCR would then be transmitted to the CD3 signaling complexes associated with the TCR through mechanisms that have yet to be discovered. Finally, a recent study showed that the juxtamembrane region of ζ–ζ homodimers are divaricated within the TCR–CD3ζ complexes and that TCR engagement drives the intra-complex juxtaposition of the ζ–ζ juxtamembrane regions (57). This mechanical switch might thus couple TCR engagement with CD3ζ-dependent signaling.

### Forces Exerted on Integrin/Ligand Bonds

Integrins are heterodimeric transmembrane proteins that mediate interactions in-between cells and interactions between cells and the extracellular matrix. Avidity of integrins is regulated by changing their valency, i.e., by changing their density at the cell/cell interface and/or changing their affinity for ligands (58). The LFA-1 integrin plays an essential role for T lymphocyte trafficking, immune synapse formation, and T lymphocyte activation (59). In resting T lymphocytes, LFA-1 is in an inactive bent conformation state, which binds with low avidity to its ligand ICAM-1. TCR stimulation induces a change in LFA-1 conformation, resulting in a more extended conformation of the integrin with an intermediate affinity (60). Finally, binding of LFA-1 to its ligand modifies further its conformation with further increase in its affinity (61, 62). Forces have been shown to play a role in affinity maturation of integrins. Indeed, application of tensile forces on integrin/ligand bonds increases bond strength and longevity (63). This was also reported for LFA-1/ICAM-1 interactions, indicating that, as for TCR/pMHC, these molecules form catch bonds (64). Moreover, it has been shown that the integrin bonds “remember” the history of the forces they have been submitted to. This phenomenon was called “cyclic mechanical reinforcement,” as the bond strength accumulates over repeated cycles of forces and is maintained after force removal (65). For instance, fibronectin/α_5_β_1_ integrin bonds dissociate within 1 s at a force of 5 pN, while upon cyclic mechanical reinforcement, the bond lifetimes can be extended to 14 s. Similar mechanisms apply to LFA-1/ICAM-1-specific bonds (65). Although head rearrangements of integrins are induced by ligand binding, this might take seconds to happen in the absence of force (66). Application of forces on the bonds would thus shorten the time required for conformational change. Moreover, cyclic mechanical reinforcement would strengthen the bonds by easing and accumulating the reversible conformational change of integrins with multiple force cycles. Therefore, during immune synapse formation, dynamic cyclic traction forces are exerted on LFA-1/ICAM-1 bonds by cycles of membrane undulations, protrusions, and retractions or by direct LFA-1 engagement, since, as described above, this can lead to force generation in T lymphocytes. By inducing conformational changes of integrins, forces during immune synapse formation can facilitate adhesion between T lymphocytes and APCs and probably participate to the costimulatory activity of LFA-1, although this remains to be tested.

## The Actomyosin Cytoskeleton: A Force Generator at the Immune Synapse

Forces experienced by T lymphocytes during synapse formation can come from the exterior but can also come from the interior generated by the cell’s own cytoskeleton. Many reviews have described and discussed remodeling of the cytoskeleton at the immune synapse and its potential role. We will herein concentrate on the role of the actomyosin cytoskeleton on the generation of forces. In the first dynamic study of immune synapse formation on artificial lipid bilayer, Grakoui et al. proposed a model of synapse formation in three stages (10): in the first stage, LFA-1 binding in the center of nascent synapse would provide “a fulcrum for cytoskeletal protrusive mechanisms that force an outermost ring of T cell membrane into close apposition with the substrate”; in the second stage, the transport of TCR–pMHC pairs to the center of the synapse would be actin driven; and in the last stage, the forces exerted would equilibrate, leading to stabilization (10). This model already proposed that forces generated by the T lymphocyte cytoskeleton would play a key role in immune synapse formation. It is remarkable to note that this model fitted so well to the experimental data obtained later on. Actin cytoskeleton has long been known to control T lymphocyte activation at different levels, such as adhesion to APC, early signaling through the TCR, and release of cytolytic granules or cytokines (67–71). T lymphocytes, when activated by the TCR–CD3, spread rapidly (in 2–4 min) on the activating substrate or cell they interact with, they stabilize (for 15–20 min), and then retract (10, 21, 72–74). These phases are reminiscent of the phases observed when adherent cells spread on their substrate (75). Indeed, the different zones of the immune synapse or supramolecular activation clusters (SMACs) have been compared to the lamellipodium (for the distal SMAC), the adhesive lamella (for the peripheral SMAC), and the uropod (for the non-adhesive central SMAC) of a mobile adherent cell (76). During synapse formation, microclusters of receptors form in the periphery and then move toward the center of the synapse (77). LFA-1 clusters stop in the pSMAC lamella zone, whereas TCR microclusters follow their path toward the cSMAC where they are endocytosed (78, 79) or secreted (80).

**Figure 2:**
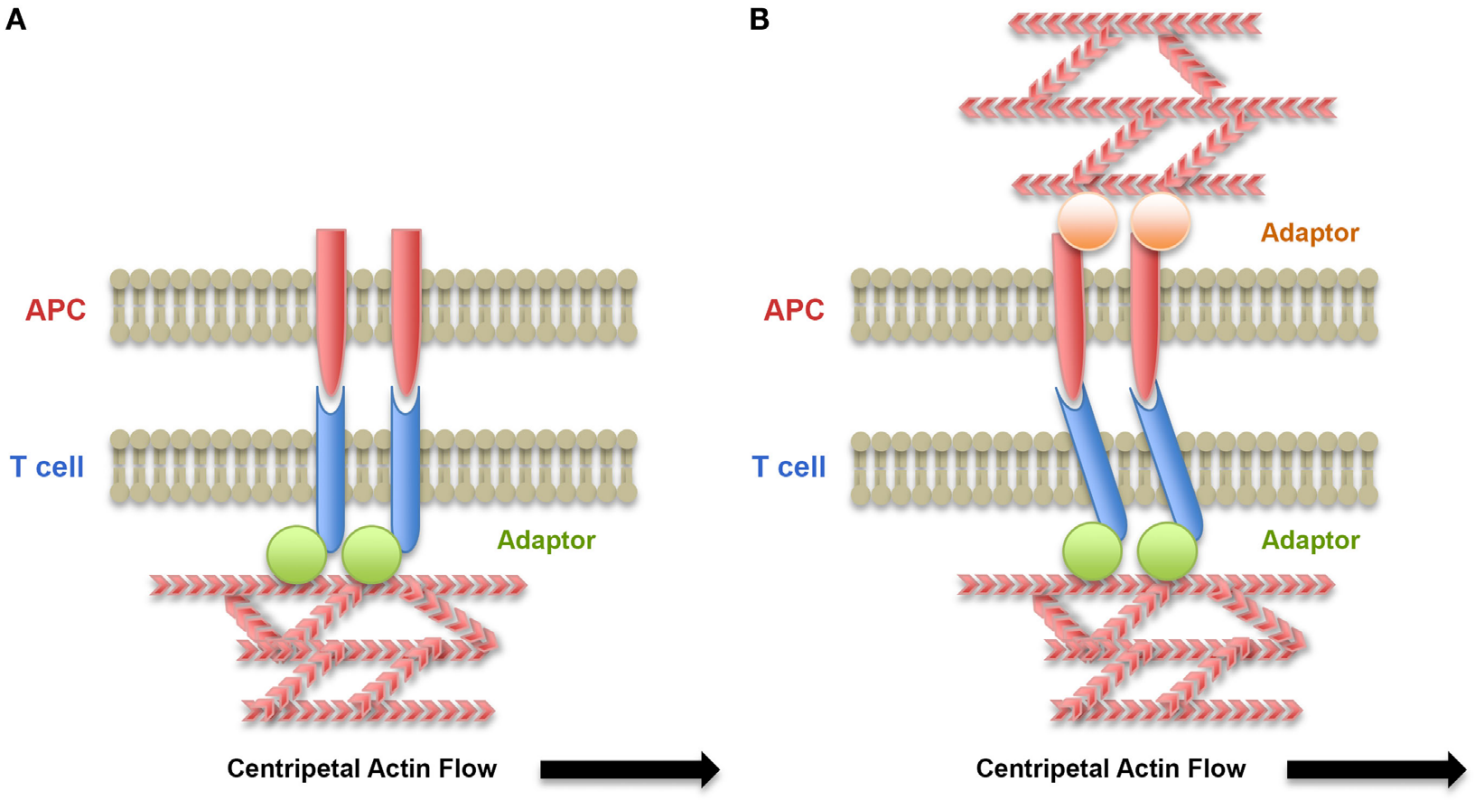
**Role of the cytoskeleton of the T lymphocyte and the APC on force exertion on receptor/ligand bonds**. **(A)** Centripetal flow of actin exerts forces on receptor/ligand bonds when receptors are coupled by an adaptor to the cytoskeleton. These forces may lead to conformational changes of the receptors and signaling. **(B)** When ligands are associated with the APC cytoskeleton, forces on receptor/ligand bonds are submitted to resistance due to reduced mobility of the ligands on the APC surface and the forces exerted on bonds are increased.

In the context of spreading described earlier, the centripetal movement of receptor clusters has been proposed to be driven by a combination of pushing forces originating from actin retrograde flow in the lamellipodium and pulling forces generated in the lamella by myosin-based contraction. Indeed, the inward flow of cortical F-actin at the immune synapse has been shown to be the major driving force behind microcluster movement (67, 81–84). The role of myosin II-based contractions at the lamella in microcluster movement, although more controversial (85), has also been shown to control the centripetal movement of both TCR and LFA-1 microclusters (86–88). One can speculate that the resistance of TCR, LFA-1, and other receptors to this mobilization would generate traction forces on the receptor/ligand bonds. Thus, coupling of receptors with the actin cytoskeleton together with mobility of the ligands at the membrane of the APC would be key elements in force generation on receptors. Adaptor molecules, such as talin, mediate interaction of LFA-1 with the actin cytoskeleton (89). The generation of localized traction forces by actin retrograde flow has indeed been shown to regulate adhesion (90, 91) in many cell types, including T lymphocytes forming immune synapses (92). In contrast, coupling of TCR to the actin cytoskeleton remains elusive. Yet, interactions of TCR clusters with actin have been revealed in experiments that introduced selective barriers, which altered TCR microcluster transport to the central SMAC (82, 93). Association of signalosomes with tyrosine-phosphorylated CD3 complexes may contribute to dynamic coupling of TCR–CD3 complexes with actin flow. The mobility of ligands on the surface of APC is another parameter to take into account into the generation of forces on receptor/ligand bonds (92, 94, 95) (Figure 2 and see later discussion in Section “T Lymphocytes Interact with Cells That Have Different Mechanical Properties”). More studies and modeling analysis are required to address these specific aspects.

## Effect of Substrate Stiffness on Force Development and T Cell Activation

The mechanical behavior of solid materials, such as plastic and glass, can be described as purely elastic. This means that their stiffness can be expressed as the ratio of the applied stress and the resulting deformation, which is termed elastic (or Young’s) modulus. On the other hand, cells and tissues display viscous properties in addition to their elastic ones and are, hence, viscoelastic materials. Two components can describe the mechanical properties of viscoelastic materials, one elastic and the other viscous, referred to as storage and loss moduli. In viscoelastic materials, the duration of force/stress application is also important, resulting in time-dependent deformations. Storage and loss moduli are different from the elastic or Young’s modulus that is more often reported in literature, since calculation of the latter is not taking into account the duration of force application. Henceforth, we refer to elastic modulus as a measure of stiffness, unless otherwise mentioned.

The effects of substrate stiffness can be as diverse as growth, differentiation, migration, and survival (96–99). Particularly, it was demonstrated that cells display differential spreading (100), velocities (101), traction forces (102), and physiological behavior (103) in response to variations in stiffness. In a seminal study (104), Discher and co-workers showed that stem cell fate could be influenced just by the stiffness of culture substrates. Another important observation was that cells match their stiffness to that of the environment by regulating their actin cytoskeleton (105). Moreover, several cell types have been reported to display durotaxis, i.e., migration from soft toward stiff substrates (101, 106).

In this section, we will report on recent studies that have begun to shed light on the mechanical properties of T lymphocyte environment and on T cell responses to these properties.

### T Lymphocytes Interact with Cells That Have Different Mechanical Properties

T lymphocytes are mobile cells that are exposed to different chemical and mechanical environments. Inside lymph nodes, T lymphocytes interact transiently with a number of different APCs, each of them potentially activated by different stimuli and presenting a varying repertoire of agonist/non-agonist peptides on MHC molecules (5, 107). In blood vessels, T lymphocytes interact with endothelial cells, and inside tissues, effector T lymphocytes interact with their targets, i.e., infected or tumor cells. Much of the studies on immune synapse formation and T cell activation have been performed on plastic or glass surfaces or on planar lipid bilayers supported on glass. Even though these surfaces provide an ideal substrate to follow receptor/ligand interactions and rearrangements, they are flat and rigid with no topological variation. Moreover, plastic and glass display stiffness in the ranges of gigapascal. In contrast, cells in the body generally display stiffness in the range of 50 Pa–40 kPa (97) with primary human T lymphocytes and Jurkat cells being at the soft end of this range (108, 109) with their stiffness ranging from 50 to 90 Pa. Therefore, in order to really study the effect of mechanical properties on T lymphocyte biology, it is vital to know the mechanical landscape that the cells encounter *in vivo* and use substrates with stiffness values inside this physiological range. Using a single-cell rheometer (110), we recently showed that different human myeloid APCs have different viscoelastic properties and that their Young’s modulus values vary from 500 Pa for monocytes and DCs to 900 Pa for macrophages (109). Moreover, inflammatory conditions modified the viscoelastic properties of myeloid cells, which were halved or doubled when cells were treated with a TNFα/PGE_2_ cocktail or IFNγ, respectively (109). These results suggest that viscoelastic properties of myeloid cells are additional parameters of inflammation that can be integrated with biochemical factors to generate an adapted T lymphocyte response. Other studies have also reported variations in myeloid cell mechanical properties (111–113). Finally, it is worth noting that endothelial cells have also been shown to change their viscoelastic properties in response to inflammation (35), suggesting that this might be a more general process.

In our study, the viscoelastic properties of human myeloid cells were dependent on myosin IIA activity and correlated to the F-actin content in each type of cells (109). These results suggest that the actomyosin cytoskeleton of myeloid cells is responsible for their mechanical properties. Interestingly, older reports have shown that DC cytoskeleton was indispensable for priming of T cells since following DC treatment with actin depolymerizing drugs, naïve CD4^+^ T cells were unable to proliferate (114). DCs were shown to polarize their cytoskeleton toward the immune synapse only upon successful antigen recognition by the T cell, and this was critical for TCR triggering and IL-2 production (115, 116). Maturation of DCs has also been associated with remodeling of their cytoskeleton, leading to development of projections directed toward T lymphocytes to optimize cell/cell interactions (117, 118). More recently, it has been shown that the cortical actin network of DCs regulated ICAM-1 lateral mobility at the cell surface and that DC maturation regulated mobility and clustering of ICAM-1 (95). The constrained ICAM-1 mobility associated with DC maturation was shown to promote formation of T lymphocyte/DC conjugates as well as T lymphocyte proliferation. On the T lymphocyte side, it was shown that LFA-1 affinity maturation correlated to ICAM-1 lateral mobility on the DC surface, i.e., low mobility of ICAM-1 induced high-affinity conformational changes of LFA-1 (95). The same group showed that actin flow in T lymphocytes was indispensable to maintain LFA-1 in the high-affinity conformation at the immune synapse and that ICAM-1 mobility directly affected distribution of high-affinity LFA-1 on the surface of engaged T lymphocytes (92). These results suggest a model in which ICAM-1 mobility on APC surface modulates resistance to tensile forces applied by the T lymphocyte actin cytoskeleton on LFA-1/ICAM-1 bonds, highlighting the role of mechanotransduction in cell conjugate formation and T lymphocyte activation (Figure 2). These mechanisms may apply to other receptor/ligand pairs, such as CD28/CD80-CD86, which are also coupled to the actin cytoskeleton (119–122). They may also apply to other cell types, such as endothelial cells. Therefore, these studies show that APCs can contribute both biochemical and mechanical cues to T cell activation. Overall, the above findings are (1) highlighting the requirements for APC/T lymphocyte crosstalk (123) for immune synapse formation and T lymphocyte activation and the need for more studies focusing on the mechanical properties of both sides of the immune synapse and (2) stressing the importance for T lymphocytes to sense the mechanical and topological properties of their environment in order to locate a specific target and respond.

It is worth noting that the mechanical properties of tissues and organs can be also modified in normal and pathological conditions. For example, it was recently shown that the contractility of fibroblastic reticular cells is regulated upon inflammation by the expression of CLEC-2 on mature dendritic cells (124, 125). CLEC-2, by interacting with podoplanin expressed on fibroblastic reticular cells, induces the relaxation of these cells that leads to a decrease of the lymph node stiffness that is probably important for its expansion (125). Moreover, tumor mechanics, and in particular the rigidity of tumoral tissues, has been shown to play a role in tumor development (126). These changes in mechanical properties of tissues and organs might also affect overall T lymphocyte activity.

Finally, it is possible that viscoelastic properties of T lymphocytes themselves are also modified by activation. The strength of TCR signaling may induce changes in T lymphocyte stiffness, which in turn may affect their interactions with APC and target cells as well as their migratory properties. Along this line, it is worth noting that T lymphocytes can adopt two types of migratory behavior (5, 127). Strong TCR stimulation can lead to complete arrest of T lymphocyte migration and stable conjugation with an APC, which can last several hours (128), while when interacting with TCR ligands of low potency or low affinity, T lymphocytes do not completely stop migrating and establish brief dynamic contacts with the APC (129), termed kinapses (127). TCR signaling strength modifies the actomyosin cytoskeleton of T lymphocytes (130, 131), which may lead to an alteration of their mechanical properties. It would thus be interesting to measure the effect of TCR signaling strength and also cytokine environment on T lymphocyte viscoelastic properties.

### T Lymphocytes Sense and Adapt to Substrate Stiffness

As discussed above, forces exerted by T lymphocytes may be important to probe their environment and particularly to test the stiffness, as we do when exerting pressure with our finger on a substrate. For example, it was proposed that “T lymphocytes are guided by the mechanical ‘path of least resistance’ as they transverse the endothelium” (34). In fact, T lymphocytes develop ILPs that physically push against the endothelial cell surface (20, 33, 34), suggesting that the role of these protrusions is to test the stiffness of endothelial cells in order to find “soft” areas to cross through (35). It is worth noting that these protrusions have also been proposed to facilitate the activation of memory/effector T cells to pMHC exposed on endothelial cells (22). Thus, T lymphocytes can sense the stiffness of the substrate they interact with. We have shown that not only T lymphocytes sense stiffness but also adapt to it. The pulling forces exerted by T lymphocytes upon TCR–CD3 triggering increased with the stiffness of the BFP used (11). This adaptation of forces to stiffness was not found in another study (12). Yet, the stiffness range used in each study might be very different.

Recent studies have addressed the effect of substrate stiffness on T lymphocyte activation. Using polyacrylamide gels with varying stiffness (range from 2 to 200 kPa) coated with an activating anti-CD3ϵ antibody, it was shown that mouse naïve CD4^+^ T lymphocytes modulated their response according to the stiffness of gel substrates (132). Production of IL-2 and early phosphorylation of Zap70 and Src family kinases was higher on “stiff” (100–200 kPa) substrates. This response to substrate stiffness was observed only when the anti-CD3ϵ antibody was attached to the gel and was abrogated in the presence of the myosin inhibitor blebbistatin (132). These results suggest that the mechanotransduction involved in T cell activation requires coupling of the TCR–CD3 to the substrate and intact myosin II activity. In another study, human naïve CD4^+^ and CD8^+^ T lymphocytes were cultured on poly-dimethoxysilane (PDMS) substrates with stiffness ranging from 100 to 10,000 kPa. The “soft” substrates (~100 kPa) induced higher IL-2 and IFNγ production as well as more T lymphocyte proliferation (133). These results seem inconsistent with the previous study (132). Yet, for poly-acrylamide gel substrates, immobilization was performed by coupling of biotinylated activating antibodies on acrylamide-conjugated streptavidin. In contrast, coating of PDMS substrates was performed by passive adsorption of antibodies on the hydrophobic surface (133), possibly resulting in both loss of immobilized material over time and passive adsorption of proteins from the culture medium. In a third study, human CD4^+^ T lymphoblasts were activated on PDMS substrates of varying stiffness, which presented anti-CD3 antibodies either alone or together with ICAM-1 molecules (134). In this study, “soft” (5 kPa) substrates induced less tyrosine phosphorylation than the “rigid” (2000 kPa) ones, and ICAM-1 increased the response to “stiff” substrates (134).

Even though the aforementioned reports provide very interesting results, their focus is on a stiffness range (2–10,000 kPa) that is non-physiological for T cells in the body, since APCs were shown to display stiffness ranging from 0.19 to 1.45 kPa (109). A recent study (13) looked at the response of Jurkat cells to substrates of a more physiologically relevant stiffness range (0.2–6 kPa). The authors used polyacrylamide gels, treated with hydrazine hydrate and coated with poly-l-lysine and an activating anti-CD3 antibody. They quantified the effect of substrate stiffness on CD3-induced signaling by following tyrosine phosphorylation by immunoblotting and microscopy (13). They showed that tyrosine phosphorylation peaked higher and more rapidly on “stiff” gels (5 kPa) but decreased more rapidly than on “soft” gels (1 kPa).

Although these studies (summarized in Table 1) are difficult to directly compare because they use different cell types, substrate chemistry, antibody immobilization, and stiffness ranges, they overall reveal that T lymphocytes are indeed mechanosensitive. It is not entirely clear what is the mechanosensing mechanism, yet, as summarized above, it requires TCR-dependent actomyosin remodeling. One explanation for the effects on TCR triggering and subsequent activation could be the local spreading and deformation of the T cell membrane in contact with substrate of different stiffness. In the kinetic-segregation model (37), local membrane deformation and segregation of large glycoproteins have to occur before TCR and pMHC can come close to one another and interact (40). It was proposed that this process required cytoskeleton-derived force (135). One could postulate that this could occur at the tips of ILPs or other small protrusions of the cell, i.e., short filopodia. Regarding the deformability of the substrate, “stiff” substrates would allow more deformation of the T lymphocyte membrane and better molecular segregation at the T cell protrusions compared to “soft” substrates and this, in turn, would result in an increased number of successful interactions between TCR and ligands (anti-CD3 antibodies or pMHC complexes). The increased number of TCR engagements would induce increased intracellular signaling that would then activate the actin cytoskeleton to produce larger cell protrusions and generate forces (11–13). This process can eventually result in increasing bond lifetimes of TCR and LFA-1 for their ligands. By inducing more conformational changes, it would lead to increased T lymphocyte activation. Thus, “stiff” substrates would display a kind of mechanical signal amplification. This mechanism has already been proposed for fibroblast adhesion on substrates of varying stiffness (100). Further work will be required to test this hypothesis for T lymphocytes.

**Table 1:** **T lymphocyte response to substrate stiffness**.

Cell type	Substrate chemistry	Activators coated	Stiffness range	T cell functions measured	Response to stiffness	Reference
Mouse naïve CD4^+^ T cells	Polyacrylamide gels containing streptavidin	Biotinylated anti-CD3, anti-CD28	10–200 kPa	IL-2 production, phosphorylation of SFK and Zap70	↑ activation with ↑ stiffness	Judokusumo et al. (132)
Human naïve CD4^+^ and CD8^+^ T cells	PDMS, passive adsorption of proteins	Anti-CD3, anti-CD28	100 kPa–2 MPa	IL-2 and IFNγ production, cell proliferation	↑ activation with ↓ stiffness	O’Connor et al. (133)
Jurkat T cells	Polyacrylamide gels treated with hydrazine hydrate	Poly-l-lysine and anti-CD3	1–5 kPa	Phosphorylation of Zap70, Lat, SLP76	↑ peak activation with ↑ stiffness, ↑ sustained activation with ↓ stiffness	Hui et al. (13)
Human primary CD4^+^ T cell blasts	PDMS, passive adsorption of proteins	Anti-CD3, ICAM-1-Fc	5 kPa–2 MPa	Tyrosine phosphorylation	↑ activation with ↑ stiffness	Tabdanov et al. (134)

## Conclusion and Perspectives

Recent evidence has shown that TCR signaling and T lymphocyte activation are not solely regulated by chemical signals of the environment but also by mechanical cues. Forces exerted by the exterior or the T lymphocyte itself regulate the lifetime of receptor/ligand bonds. This, in turn, increases adhesion of T lymphocytes to APCs and allows for better discrimination of agonist pMHC. Forces exerted by T lymphocytes also help the cells probe the substrates they interact with by testing their stiffness, which might be a key parameter of T cell activation. We now need to explore further the viscoelastic properties of cells and tissues in physiological and pathological conditions in order to develop experimental models that better mimic the mechanical landscape of T lymphocytes. At the molecular level, we need to study the role of known costimulators or co-inhibitors of T lymphocyte activation in force development and force sensing and find out potential mechanical crosstalk between receptors. Finally, at the cell level, it would be interesting to study if and how mechanical cues can modulate the functions of different T lymphocyte subsets. It would be particularly important to see if mechanics can modulate naïve T lymphocyte priming or effector T lymphocyte functions (cytotoxicity and cytokine secretion). Elucidating these issues will provide further insight into T lymphocyte activation under normal and pathological conditions that could be translated in novel therapeutic strategies.

## Author Contributions

CH wrote the review, edited the manuscript, and designed table and figures. MS wrote part of the review and edited the manuscript and figures and table.

## Conflict of Interest Statement

The authors declare that the research was conducted in the absence of any commercial or financial relationships that could be construed as a potential conflict of interest.
